# The effects of prehabilitation on body composition in patients undergoing multimodal therapy for esophageal cancer

**DOI:** 10.1093/dote/doac046

**Published:** 2022-07-07

**Authors:** Laura J Halliday, Piers R Boshier, Emre Doganay, Venetia Wynter-Blyth, John P Buckley, Krishna Moorthy

**Affiliations:** Department of Surgery and Cancer, Imperial College London, UK; Department of Surgery and Cancer, Imperial College London, UK; Department of Surgery and Cancer, Imperial College London, UK; Oesophago-Gastric Cancer Surgery Unit, St Mary’s Hospital, Imperial College Healthcare NHS Trust, London, UK; Centre for Active Living, University Centre Shrewsbury/University of Chester, Shrewsbury, UK; Department of Surgery and Cancer, Imperial College London, UK

**Keywords:** adiposity, cancer, preoperative exercise, sarcopenia

## Abstract

Prehabilitation aims to optimize a patient’s functional capacity in preparation for surgery. Esophageal cancer patients have a high incidence of sarcopenia and commonly undergo neoadjuvant therapy, which is associated with loss of muscle mass. This study examines the effects of prehabilitation on body composition during neoadjuvant therapy in esophageal cancer patients. In this cohort study, changes in body composition were compared between esophageal cancer patients who participated in prehabilitation during neoadjuvant therapy and controls who did not receive prehabilitation. Assessment of body composition was performed from CT images acquired at the time of diagnosis and after neoadjuvant therapy. Fifty-one prehabilitation patients and 28 control patients were identified. There was a significantly greater fall in skeletal muscle index (SMI) in the control group compared with the prehabilitation patients (Δ SMI mean difference = −2.2 cm^2^/m^2^, 95% CI –4.3 to −0.1, *p*=0.038). Within the prehabilitation cohort, there was a smaller decline in SMI in patients with ≥75% adherence to exercise in comparison to those with lower adherence (Δ SMI mean difference = −3.2, 95% CI –6.0 to −0.5, *P* = 0.023). A greater decrease in visceral adipose tissue (VAT) was seen with increasing volumes of exercise completed during prehabilitation (*P* = 0.046). Loss of VAT during neoadjuvant therapy was associated with a lower risk of post-operative complications (*P* = 0.017). By limiting the fall in SMI and promoting VAT loss, prehabilitation may have multiple beneficial effects in patients with esophageal cancer. Multi-center, randomized studies are needed to further explore these findings.

## INTRODUCTION

Cachexia and sarcopenia are common features of esophageal cancer,^1^ characterized by dysregulated energy metabolism and wasting of skeletal muscle. Predisposing factors include the burden of the tumor itself and the physiological effects of chemoradiotherapy,^2^^,^^3^ exacerbated by anorexia and mechanical obstruction of the esophagus. Previous studies have identified sarcopenia as a negative prognostic indicator in patients with esophageal cancer, predicative of chemotherapy toxicity, postoperative complications and worse overall survival.^1^^,^^2^^,^^4–6^

Prehabilitation is an emergent field of perioperative medicine focusing on strategies to optimize a patient’s functional capacity in preparation for the physiological challenge of major surgery. Although highly variable, common components of prehabilitation programs include physical exercise, nutrition and psychological interventions.^7^ In patients undergoing major intra-abdominal surgery, prehabilitation has been associated with a 40% reduction in overall postoperative complications and a 60% reduction in pulmonary complications.^8^^,^^9^

Esophageal cancer patients are notable for their high incidence of sarcopenia.^1^ This is compounded by the common requirement for neoadjuvant therapy, which is itself associated with a fall in muscle mass.^3^^,^^10^ Preoperative exercise may therefore have a particularly beneficial effect on muscle mass in this high-risk group of patients. Prehabilitation has been shown to reduce skeletal muscle loss in patients with esophageal cancer during neoadjuvant therapy and increase skeletal mass in rectal cancer patients undergoing neoadjuvant chemoradiotherapy.^11^^,^^12^ However, the effects of prehabilitation on other body composition parameters have not been studied to date.

The primary aim of this exploratory study was to assess the impact of prehabilitation on changes in body composition in patients undergoing multimodal treatment for esophageal cancer. Secondary aims of the study include identifying factors that are associated with changes in body composition in prehabilitation patients and examining the relationship between changes in body composition and postoperative outcomes.

## METHODS

### Study design

This was a single-center retrospective cohort study. Ethical approval for retrospective analysis of patient data was granted by the UK Health Research Authority (ref: 268837).

### Subjects

Patients who underwent esophagectomy after neoadjuvant therapy for esophageal or gastro-esophageal junction (GOJ) cancer at Imperial College NHS Healthcare Trust between January 2015 and December 2018 were eligible for inclusion in this study.

From January 2016 to December 2018 all patients were invited to participate in a structured prehabilitation program. Patients who completed this program were included in the prehabilitation study group. Patients who did not complete or declined the program were excluded from this study. The control group comprised of patients who received treatment in 2015 (prior to the introduction of the program) and patients from 2016 to 2018 who underwent resection at the same center as the prehabilitation cohort but either started their treatment at a different center so did not participate in the program or were not included in the prehabilitation program due to administrative constraints during the initial introduction of the program. Other than the provision of prehabilitation, the perioperative care of all patients was comparable.

### PREPARE for Surgery prehabilitation program

The PREPARE for Surgery (Physical activity, Respiratory exercises, Eat well, Psychological well-being, Ask about medications, Remove bad habits, Enhanced recovery) prehabilitation program was developed as a quality improvement initiative. Details of the PREPARE for Surgery program have previously been published^13^ and are summarized below.

PREPARE for Surgery is a home-based, multimodal prehabilitation program, which starts immediately after the completion of staging investigations and continues throughout neoadjuvant therapy until the time of surgery (covering a period of approximately 16 weeks).

### Exercise intervention

Patients were prescribed a personalized exercise program by a trained exercise therapist. This included a combination of aerobic and strength exercises with a prescribed frequency, intensity and duration for each exercise .^14^ The type, frequency, intensity and duration of each exercise was personalized according to the results of submaximal exercise testing, activities of daily living, previous exercise behavior, medical co-morbidities and social circumstances. An example of a personalized exercise prescription is provided in Supplementary File 1.

Weekly telephone ‘touch-points’ with an exercise therapist were used to monitor adherence. Providing the patient was achieving their exercise prescription, the program was increased by frequency, time and then intensity. In keeping with WHO guidelines, patients were prescribed a minimum of 600 metabolic equivalent of task (MET) minutes week^−1^ (150 minutes of moderate intensity activity), with the aim of increasing this to 1200 MET minutes week^−1^ (300 minutes of moderate intensity activity per week).^15^

Using exercise diaries, patients self-reported the frequency, duration and intensity with which they completed each exercise every week. To self-regulate and assess the intensity of exercise, patients were trained to use the Borg scale rating of perceived exertion (RPE).^16–18^ RPE scores were used to estimate the percentage of METSmax at which they exercised.^18^ At the start of the program each patient’s METSmax was calculated using the Chester Step Test;^19^^,^^20^ thus, using the METSmax and the percentage derived from the RPE scores, the estimated achieved intensity in METS was calculated. The weekly exercise duration, intensity and frequency for each activity were multiplied to provide an estimate of the volume of physical activity in MET minutes week^−1^.

There is no standardized method for measuring adherence in exercise studies.^21^ A weekly adherence was calculated by dividing the self-reported completed volume of physical activity in MET minutes week^−1^ by the prescribed MET minutes week^−1^, expressed as a percentage. There is no definition for acceptable levels of adherence to exercise.^21^ Acceptable adherence to the prescribed exercise program was pragmatically defined as an average weekly adherence of 75% or greater across the whole program.

### Nutritional intervention

All patients were reviewed by a specialist esophagogastric cancer dietitian who undertook an assessment of nutritional status including identification and stratification of nutritional risk. Based on patients’ self-reported dietary eating habits, symptoms (such as dysphagia) and biochemical nutritional deficiencies, a personalized plan was created. ESPEN guidelines for clinical nutrition in cancer patients were used to determine estimated dietary requirements: energy intake of 25–30 kcal/kg/day, protein intake of 1.0–1.5 g/kg/day, and vitamin and mineral intake at the recommended daily allowances.^22^ Interventions included dietary advice, oral supplementation or enteral feeding via a jejunostomy or nasogastric tube. Weekly or fortnightly phone calls were used to monitor nutritional status.

### Analysis of body composition

Patient’s body composition was assessed at diagnosis (before neoadjuvant therapy) and after completion of neoadjuvant therapy (before surgery). Assessment was performed using a single contrast-enhanced CT image, taken at the midpoint of the third lumbar (L3) vertebral body. CT images were exported from the picture archiving and communication system (PACS) and saved as an anonymized Digital Imaging and Communications in Medicine (DICOM) file. Anonymization of CT images was confirmed using the MIRC DICOM Editor (Ver. 35. MIRC; http://mirc.rsna.org). Segmentation of skeletal muscle (−29 to +150 HU), visceral (−150 to −50 HU) and subcutaneous (−190 to −30 HU) adipose tissues was performed using Slice-O-Matic (Ver. 5.0, Tomovision, Magog, Canada) using the ABACS-L3 module (Ver. 1.0, Voronoi Health Analytics, Canada). Two trained assessors (LH, PB), who were blinded to patient identity and image sequence, performed subsequent manual correction of segmented images.

Skeletal muscle index (SMI) was calculated as the ratio of lumbar skeletal muscle area to height squared. Sarcopenia was defined using Prado’s criteria for low muscle mass: SMI <52.4 cm^2^/m^2^ for men and < 38.5 cm^2^/m^2^ for women.^23^ Sarcopenic obesity was defined as sarcopenia in the presence of body mass index (BMI) ≥30 kg/m^2^. Visceral obesity was defined as a visceral fat area > 163.8 cm^2^ for men and > 80.1 cm^2^ for women.^2^

### Hand-grip strength in the prehabilitation cohort

Hand-grip strength was measured as a part of the prehabilitation program to provide a validated assessment of muscle function.^24^ It was measured at the start of prehabilitation and again following completion of neoadjuvant therapy using a Takei digital hand-grip dynamometer. Patients were asked to squeeze the dynamometer as tight as possible using their non-dominant hand and the highest of three repeated readings was recorded.^25^

### Outcome measurements

The primary outcome measure of this study was change in parameters of body composition (weight, BMI, skeletal muscle, visceral adipose tissue, subcutaneous adipose tissue and total adipose tissue). Secondary outcome measures included hand-grip strength, adherence to preoperative exercise, volume of physical activity completed during prehabilitation and 60-day postoperative complications. Complications were defined according to the Esophagectomy Complication Consensus Group (ECCG) guidelines^26^ (whereby lower respiratory tract infections were defined by the American Thoracic Society guidelines for hospital acquired pneumonia^27^).

### Statistical analysis

Statistical analysis was performed using SPSS version 26 (IBM, New York, USA). Normality of data was assessed visually and using the Kolmogorov–Smirnov (with Lilliefors correction) and Shapiro–Wilk normality tests. Depending on their distribution, continuous variables are presented as either mean ± standard deviation or median [interquartile range, IQR]. Changes in continuous variables over time were assessed using a paired T test or Wilcoxon test, respectively. Between-group comparison of continuous variables was performed using the Independent-Samples T test or Mann–Whitney U test, respectively. Categorical variables were compared using the chi-squared or Fisher’s exact tests. Correlation between continuous variables was assessed using a Pearson’s or Spearman’s rank test, depending on data distribution. Multiple regression analysis was used to determine the factors associated with the change in body composition and binary logistic regression was used to determine the factors associated with postoperative outcomes. Two-tailed tests were used throughout with a significance level of *P* < 0.05.

## RESULTS

**Fig. 1:**
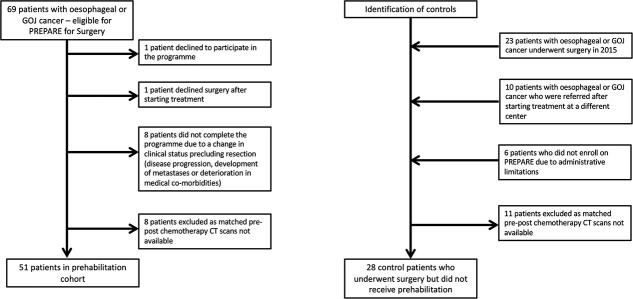
Study participant flow chart

Between January 2016 and December 2018, 69 patients with esophageal or GOJ cancer were invited to participate in the PREPARE program prior to starting neoadjuvant therapy. Eighteen patients were excluded: declined to participate in the PREPARE program (*n* = 1); declined surgery (*n* = 1); change in clinical status precluding resection (disease progression or medical co-morbidities) (*n* = 8); and lack of availability of matched CT images pre- and post-neoadjuvant therapy (*n* = 8). No patients dropped out of the prehabilitation program. Consequently, 51 patients were included in the prehabilitation group (Fig. Fig. 1). Thirty-nine control patients who underwent neoadjuvant therapy followed by surgery but did not complete prehabilitation were identified (Fig. Fig. 1). Matched CT images were not available for 11 of these patients, and therefore 28 control patients were included in the analysis. Characteristics of study participants are presented in Table 1.

**Table 1:** Study participant characteristics

	Prehabilitation patients (*n* = 51)	Controls (*n* = 28)	*P-*Value
Age (years)	66 2 ± 9.9	63.5 ± 9.6	0.245
Gender, male *n* (%)	37 (73%)	22 (76%)	0.556
Tumor location *n* (%)			
Esophagus	32 (63%)	17 (61%)	0.859
GOJ	19 (37%)	11 (39%)	
Histological subtype *n* (%)			
Adenocarcinoma	43 (84%)	21 (75%)	0.313
Squamous cell carcinoma	8 (16%)	7 (25%)	
Neoadjuvant therapy *n* (%)			
Chemotherapy	37 (73%)	21 (75%)	0.814
Chemoradiotherapy	14 (27%)	7 (25%)	
Clinical stage *n* (%)			
I	1 (2%)	1 (3%)	0.357
II	7 (14%)	3 (11%)	
II	35 (69%)	15 (54%)	
IV	8 (15%)	9 (32%)	
ASA grade *n* (%)			
II	44 (86%)	21 (75%)	0.209
III	7 (14%)	7 (25%)	
Charlson Comorbidity Index	4.7 ± 1.3	4.4 ± 1.2	0.351
Time interval between scans days	97.6 ± 26.0	119.0 ± 39.5	0.014
Esophagectomy			
2-stage	35 (68%)	15 (54%)	0.314
3-stage	9 (18%)	9 (32%)	
Thoracoabdominal	7 (14%)	4 (14%)	
Surgical approach			
Open	48 (94%)	25 (89%)	0.438
Hybrid minimally invasive ^†^	3 (6%)	3 (11%)	
Baseline body composition			
Weight (kg)	80.7 ± 19.8	79.5 ± 13.3	0.772
BMI (kg/m^2^)	27.6 ± 6.8	26.6 ± 3.5	0.394
SM area (cm^2^)	133.8 ± 32.0	149.3 ± 26.7	0.033
SMI (cm^2^/m^2^)	45.4 ± 8.9	49.9 ± 6.8	0.024
TAT area (cm^2^)	398.4 ± 192.4	403.6 ± 154.4	0.902
VAT area (cm^2^)	171.3 ± 90.3	176.0 ± 102.7	0.834
SAT area (cm^2^) ‡	200.1 (151.3, 278.8)	211.1 (165.1, 296.4)	0.580
Sarcopenia, *n* (%)	34 (66%)	14 (50%)	0.147
Visceral obesity, *n* (%)	33 (65%)	15 (54%)	0.332

Continuous data presented as mean ± SD unless otherwise stated. GOJ = gastro-esophageal junction; ASA = American Society of Anesthesiologist physical status classification; NA-therapy = neoadjuvant therapy; SM = skeletal muscle; SMI = skeletal muscle index; TAT = total adipose tissue; VAT = visceral adipose tissue; SAT = subcutaneous adipose tissue.

^†^Laparoscopic abdominal stage, open thoracic stage; ‡Non-parametric data, displayed as median (interquartile range)

Within the prehabilitation group, 18 patients (35%) received oral nutritional supplementation during neoadjuvant therapy and four (8%) received supplemental feeding by either a nasogastric tube or jejunostomy. Seven patients in the control group received oral nutritional supplementation (25%) and one patient received jejunostomy feeding (4%). There was no significant difference in the use of nutritional interventions between the two groups (oral supplementation *P* = 0.347, nasogastric or jejunostomy feeding *P* = 0.456).

### Changes in body composition during neoadjuvant therapy

Baseline body composition characteristics are presented in Table 1. A significant decrease in weight, BMI, skeletal muscle (SM) area, skeletal muscle index (SMI), visceral adipose tissue (VAT) and total adipose tissue (TAT) was observed in both groups after neoadjuvant therapy (Table 2).

**Table 2:** Change in body composition parameters during neoadjuvant therapy

	Prehabilitation patients (*n* = 51)	Controls (*n* = 28)	Mean difference (95% CI)	*P-*Value
Δ Weight (kg)	−1.6 ± 5.2^*^	−3.0 ± 4.5^*^	−1.5 (−3.8 to 0.9)	0.215
Δ BMI (kg/m^2^)	−0.6 ± 1.9^*^	−1.1 ± 1.5^*^^*^	−0.4 (−1.3 to 0.4)	0.313
Δ SM area (cm^2^)	−8.7 ± 14.0^*^^*^^*^	−15.5 ± 11.3^*^^*^^*^	−6.8 (−12.9 to −0.6)	0.031
Δ SMI (cm^2^/m^2^)	−3.0 ± 4.8^*^^*^^*^	−5.2 ± 3.7^*^^*^^*^	−2.2 (−4.3 to −0.1)	0.038
Δ TAT area (cm^2^)	−27.3 ± 81.0^*^^*^^*^	−33.3 ± 63.2^*^^*^	−6.0 (−41.2 to 29.2)	0.735
Δ VAT area (cm^2^)	−16.9 ± 48.0^*^	−19.4 ± 42.4^*^	−2.5 (−24.1 to 19.1)	0.818
Δ SAT area (cm^2^) ^†^	−5.6 (−31.0, 16.9)	−11.0 (−35.7, 4.8)	n/a	0.608
Relative changes in body composition				
Relative Δ Weight (%)	−1.5 ± 6.5	−3.9 ± 5.9	−2.5 (−5.3 to 0.5)	0.099
Relative Δ BMI (%)	−1.7 ± 7.0	−3.9 ± 5.9	−2.3 (−5.4 to 0.8)	0.151
Relative Δ SM area (%)	−6.1 ± 11.4	−10.6 ± 7.5	−4.5 (−8.7 to −0.2)	0.039
Relative Δ SMI (%)	−6.3 ± 11.6	−10.6 ± 7.5	−4.3 (−8.5 to −0.2)	0.050
Relative Δ TAT area (%) †	−6.1 (−21.2, 6.4)	−9.4 (−19.8, −0.1)	n/a	0.559
Relative Δ VAT area (%) †	−10.7 (−28.9, 2.9)	−12.4 (−30.5, 0.9)	n/a	0.731
Relative Δ SAT area (%) ^†^	−3.9 (−18.0, 8.1)	−5.8 (−14.9, 1.6)	n/a	0.678


Continuous data presented as mean ± SD unless otherwise stated. SM = skeletal muscle; SMI = skeletal muscle index; TAT = total adipose tissue; VAT = visceral adipose tissue; SAT = subcutaneous adipose tissue; n/a = not applicable.

^†^Non-parametric data, displayed as median (interquartile range).

Within group comparison of change before and after neoadjuvant therapy: ^*^P < 0.05; ^*^^*^P < 0.01; ^*^^*^^*^P < 0.001

Comparing the change in body composition between the two groups, there was a significantly greater fall in SMI in the control group compared with the prehabilitation patients (Table 2; Δ SMI mean difference = −2.2 cm^2^/m^2^, 95% CI -4.3 to −0.1, *P* = 0.038). There was also a larger % decrease in SMI in the controls compared with the prehabilitation patients (Table 2; relative Δ SMI mean difference = −4.3%, 95% CI –8.5 to −0.2, *P* = 0.05). There were no significant differences in changes in other body composition parameters between the two groups.

To adjust for the lower baseline SM area and SMI in the prehabilitation group, a propensity score was created using a multivariate logistical regression model, with SM area and SMI as co-variates. Using the propensity score, patients in the prehabilitation group were matched 1:1 to those in the control group, with a match tolerance of 0.05. This generated 28 patients in each group (Supplementary File 2). Both the absolute and relative changes in SMI remained significantly different between the two groups, with a larger fall in the control group (Supplementary File 2).

### Hand-grip strength

Hand-grip strength was measured in the prehabilitation group only. It did not vary significantly between assessment at diagnosis and after neoadjuvant therapy (30.7 ± 8.5 vs. 30.3 ± 8.3; *P* = 0.491). Hand-grip strength at diagnosis (*R*^2^ = 0.576, *P* = 0.001) and after neoadjuvant chemotherapy (*R*^2^ = 0.554, *P* = 0.001) was correlated to skeletal muscle area.

### Change in body composition and adherence to pre-operative exercise in prehabilitation patients

Data on exercise adherence and physical activity during prehabilitation was available for 47 patients in the prehabilitation cohort (92%). The mean amount of activity completed during neoadjuvant therapy was 858 ± 727 MET minutes week^−1^. The mean adherence to the personalized exercise prescriptions during neoadjuvant therapy was 55 ± 31.3%.

Variations in body composition parameters based on patient adherence to the personalized exercise prescriptions are presented in Table 3. The decline in SMI was significantly less in patients with ≥75% adherence (Δ SMI mean difference = −3.2 cm^2^/m^2^, 95% CI –6.0 to −0.5 *P* = 0.023). There were no significant differences in the changes in other body composition parameters between patients with ≥75% and < 75% adherence. When the adherence threshold was decreased to ≥50%, compliance with the prescribed exercise program was no longer protective for preservation of the SM area and SMI (Table 3).

**Table 3:** Change in body composition parameters stratified by adherence to personalized exercise prescriptions

	Adherence ≥ 75% *n* = 15	Adherence < 75% *n* = 32	Mean difference (95% CI)	*P-*Value
Δ Weight (kg)	0.3 ± 5.9	−2.2 ± 4.5	−2.4 (−6.0 to 1.1)	0.169
Δ BMI (kg/m^2^)	0.1 ± 1.9	−0.9 ± 1.9	−1.0 (−2.1 to 0.2)	0.105
Δ SM area (cm^2^)	−1.4 ± 13.3	−10.3 ± 12.3	−8.9 (−16.9 to −0.9)	0.040
Δ SMI (cm^2^/m^2^)	−0.4 ± 4.2	−3.7 ± 4.4	−3.2 (−6.0 to −0.5)	0.023
Δ TAT area (cm^2^)	−32.0 ± 106.9	−31.5 ± 71.6	0.5 (−63.0 to 64.0)	0.987
Δ VAT area (cm^2^)	−14.4 ± 65.4	−22.2 ± 39.2	−7.8 (−38.9 to 23.3)	0.616
Δ SAT area (cm^2^) †	−5.6 (−25.8, 12.5)	−10.2 (−40.6, 16.9)	n/a	0.916
Δ Hand-grip strength (kg)	−0.2 ± 3.2	−0.2 ± 3.5	n/a	0.970
	Adherence ≥ 50% *n* = 28	Adherence < 50% *n* = 19	Mean difference (95% CI)	*P-*Value
Δ Weight (kg)	−0.9 ± 5.5	2.0 ± 4.3	1.2 (−1.7 to 4.0)	0.424
Δ BMI (kg/m^2^)	−0.4 ± 1.9	−0.9 ± 2.0	0.6 (−0.6 to 1.7)	0.323
Δ SM area (cm^2^)	−4.8 ± 13.3	−10.9 ± 12.2	−6.1 (−13.7 to 1.5)	0.114
Δ SMI (cm^2^/m^2^)	−1.6 ± 4.6	−3.9 ± 4.3	−2.2 (−4.9 to 0.4)	0.096
Δ TAT area (cm^2^)	−25.0 ± 95.8	−38.7 ± 60.1	−13.7 (−59.8 to 32.4)	0.552
Δ VAT area (cm^2^)	−11.7 ± 53.3	−29.5 ± 39.2	−17.7 (−44.9 to 9.5)	0.197
Δ SAT area (cm^2^) ^†^	−7.9 (−35.0, 19.0)	−9.3 (−40.6, 9.1)	n/a	0.897
Δ Hand-grip strength (kg)	−0.2 ± 2.9	−0.1 ± 4.1	n/a	0.934

Continuous data presented as mean ± SD unless otherwise stated. SM = skeletal muscle; SMI = skeletal muscle index; TAT = total adipose tissue; VAT = visceral adipose tissue; SAT = subcutaneous adipose tissue; n/a = not applicable.

^†^Non-parametric data, displayed as median (interquartile range)

On multivariate analysis, there was no relationship between the decrease in SMI during neoadjuvant therapy and age, ASA grade, Charlson Comorbidity Index (CCI), clinical stage, baseline fitness, adherence (%) or average weekly physical activity (Supplementary File 3). However, increasing amounts of average weekly physical activity were associated with a greater loss of VAT during neoadjuvant therapy (Supplementary File 3, *P* = 0.046).

### Change in body composition and postoperative outcomes

Thirty-three prehabilitation patients (65%) developed one or more postoperative complications, 13 of which were classified as severe complications (Clavien Dindo grade ≥ 3). Twenty control patients (71%) developed one or more postoperative complications, 14 of which were severe complications (Clavien Dindo grade ≥ 3). There was no significant difference in the overall complication rate between the two groups (*P* = 0.543). There was, however, a trend for a lower incidence of lower respiratory tract infection in the prehabilitation group: 17 patients (33%) in the prehabilitation group, compared with 15 patients (54%) in the control group, *P* = 0.080. Comparison of complications in the propensity score matched groups is shown in Supplementary File 2.

Patients who developed complications lost significantly less VAT during neoadjuvant therapy in comparison to patients who did not develop complications (Table 4, Δ VAT mean difference = −23.7 cm^2^, 95% CI –45.0 to −2.3, *P* = 0.030). On multivariate analysis, loss of VAT during neoadjuvant therapy was associated with a lower risk of postoperative complications (Supplementary File 3, *P* = 0.017).

**Table 4:** Variation in body composition in all study participants, stratified by postoperative complications

	Any complication *n* = 53	No complications *n* = 26	Mean difference (95% CI)	*P-*Value
Δ Weight (kg)	−2.1 ± 5.3	−2.1 ± 4.4	0.0 (−2.4 to 2.4)	0.996
Δ BMI (kg/m^2^)	−0.8 ± 1.9	−0.7 ± 1.5	0.1 (−0.8 to 1.0)	0.821
Δ SM area (cm^2^)	−11.0 ± 12.7	−11.5 ± 15.1	−0.5 (−6.9 to 6.0)	0.882
Δ SMI (cm^2^/m^2^)	−3.7 ± 4.4	−3.8 ± 4.9	−0.1 (−2.3 to 2.1)	0.935
Δ TAT area (cm^2^)	−16.2 ± 75.3	−56.4 ± 67.6	−40.1 (−74.9 to −5.4)	0.024
Δ VAT area (cm^2^)	−10.0 ± 47.2	−33.7 ± 39.4	−23.7 (−45.0 to −2.3)	0.030
Δ SAT area (cm^2^) ^†^	−2.9 (−20.3, 16.8)	−16.3 (−44.4, 0.1)	n/a	0.055

Continuous data presented as mean ± SD unless otherwise stated. SM = skeletal muscle; SMI = skeletal muscle index; TAT = total adipose tissue; VAT = visceral adipose tissue; SAT = subcutaneous adipose tissue; n/a = not applicable.

^†^Non-parametric data, displayed as median (interquartile range)

## DISCUSSION

We have observed that prehabilitation appears to preserve skeletal muscle during neoadjuvant therapy. We have also found some evidence of a dose–response effect, with greater preservation of skeletal muscle with higher levels of adherence to preoperative exercise. Finally, we have observed that loss of VAT may be protective against postoperative complications.

Our finding of a positive impact of prehabilitation on muscle mass is in keeping with previous studies; prehabilitation during neoadjuvant therapy has been shown to reduce muscle loss in esophageal cancer patients and increase skeletal mass in rectal cancer patients.^11^^,^^12^ In keeping with this previous study of prehabilitation in esophageal cancer patients,^11^ we found a similar magnitude of change in muscle mass in our study, and also found that hand-grip strength was preserved in prehabilitation patients during neoadjuvant therapy, suggesting preservation of muscle function. However, in our study hand-grip strength was not recorded in the control group, and therefore a comparison of function between the two groups was not possible.

Not all studies into the effects of prehabilitation on muscle mass have shown a beneficial effect. Studies where patients did not receive neoadjuvant therapy have failed to show a significant impact of prehabilitation on muscle mass.^28^^,^^29^ We believe that particular benefit may be derived from exercise during neoadjuvant treatment due to the deleterious effect of chemotherapy and chemoradiotherapy on muscle mass.

The prehabilitation patients in our study who had high adherence to their personalized exercise program had greater preservation of skeletal muscle. This benefit was not maintained when the threshold for acceptable adherence was decreased, highlighting the importance of regular participation in exercise to limit loss of skeletal muscle. In our multivariate analysis of factors associated with loss of skeletal muscle within the prehabilitation patients, the overall adherence percentage was not a significant factor. This may imply a non-linear relationship between exercise adherence and preservation of muscle, and high levels of adherence may be needed to limit muscle loss. More research is needed into ways to improve adherence to home-based exercise during neoadjuvant therapy, such as wearable technology, video exercises, greater exercise personalization and the incorporation of other behavior change techniques.

Overall, we saw a significant decrease in VAT during neoadjuvant therapy. A fall in VAT:SAT is seen in esophageal cancer patients during neoadjuvant therapy,^3^ indicating that changes in adiposity may occur as a consequence of the underlying disease and oncological treatments. However, we found that the amount of VAT lost in the preoperative period was significantly related to the volume of exercise completed, with a greater decrease in VAT seen with increasing volumes of physical activity.

Our study is the first to observe a relationship between changes in adiposity during neoadjuvant therapy and postoperative complications. There was a high incidence of visceral obesity in our study population. Obesity is a risk factor for developing esophageal cancer.^30–32^ It is also a poor prognostic factor in some cancers; for example, pancreatic cancer patients with a high VAT:SAT ratio have a lower overall survival and disease-free survival,^33^ and VAT:abdominal muscle area ratio is an independent risk factor for postoperative complications in patients with gastric cancer.^34^ In esophageal cancer patients, high volumes of VAT after neoadjuvant therapy is associated with poorer overall survival.^35^ VAT is metabolically active and secretes a range of pro-inflammatory cytokines.^36^ Reducing adiposity reduces systemic inflammation and improves glycemic control,^37–39^ which represents a possible mechanism by which reducing VAT may reduce the risk of postoperative complications. Further research is needed to examine these parameters in relation to changes in VAT during prehabilitation.

There is significant heterogeneity in the content of prehabilitation programs, including substantial differences in the type and intensity of exercise.^9^^,^^40^ Given the relationship we have observed between VAT loss and complications, we propose that exercise programs targeting VAT loss should be explored. High or moderate intensity exercise is associated with VAT loss in overweight and obese patients.^41^^,^^42^ High-intensity interval training (HIIT) in cancer survivors has also been shown to result in larger reductions in fat mass compared with low or moderate intensity continuous training .^43^^,^^44^ HIIT was not used in this study, but it has been trialed in patients undergoing prehabilitation and shown to be safe and feasible, with significant improvements in cardiorespiratory fitness.^45^ However, the effect of HIIT-based prehabilitation on body composition and postoperative outcomes has not been established, and the impact of HIIT has not been compared with moderate or low intensity exercise in the preoperative setting.

Both dietary modifications and exercise can be used to induce VAT loss .^46^^,^^47^ However, studies suggest that a reduction in VAT is not seen without overall weight loss when dietary interventions alone are used, whereas VAT loss can be achieved with exercise even when no significant weight loss occurs.^47^ Due to the metabolic effects of cancer, overall weight loss may not be desirable in all patients and therefore exercise may be a more suitable strategy to achieve VAT loss compared with calorie restriction.

The nutritional support needed by esophageal patients is very variable. While some patients may need to lose weight, others will need to gain weight, and some may have very poor oral intake due to dysphagia. In keeping with guidelines from Macmillan Cancer Support, nutritional support was a key component of the prehabilitation program in this study^48^ and patients were reviewed every two weeks by a specialist esophagogastric cancer dietitian, using standardized guidelines to optimize nutrition. This proactive approach contrasts with the reactive approach to nutrition in the standard preoperative cancer pathway used in the control patients. Whilst nutritional deficiencies may have been identified and addressed in the control patients, traditional preoperative nutritional support is often ad hoc, with variable nutritional input from a range of healthcare professionals. A higher proportion of patients in the prehabilitation group received oral supplementation or supplemental feeding by either a nasogastric tube or jejunostomy compared with the controls, and although these differences were not statistically significant, we believe that this reflects the proactive approach in the prehabilitation program to identifying and managing nutritional deficiencies.

There are several limitations to this study. The time interval between CT scans was longer in the control group than in the prehabilitation patients (Table 1). There are no studies to date assessing the rate of preoperative muscle mass loss in esophageal cancer patients, so it is not possible to definitively conclude what effect this time difference may have on changes in muscle mass. Nonetheless, the longer time interval in the control group may contribute to the differences in muscle loss between the two groups and it is possible that the difference in change in muscle mass may be a result of this time discrepancy. However, our finding of less muscle loss with prehabilitation is in keeping with a previous study in this patient group.^11^

All patients who completed the program between January 2016 and December 2018 and underwent neoadjuvant chemotherapy were eligible for this study. Despite this, the sample size may be insufficient to detect the effect of changes in some body composition parameters, such as SMI, on postoperative outcomes. Other factors independent of the prehabilitation program may have influenced both the changes in body composition and postoperative outcomes. This includes the use of neoadjuvant radiotherapy, the number of cycles of chemotherapy received, and side effects and toxicity from neoadjuvant therapy. Due to the sample size, it was not possible to undertake subgroup analyses to control for the effects of these factors. Furthermore, although we have reported postoperative outcomes in this study, this was not the primary outcome, and the study was not powered to detect a difference in complications. A large, multi-center randomized controlled trial should be undertaken to provide a sufficient sample size to allow the analysis of a broader range of outcomes and to control for the effects of different patient variables. Multi-center studies are also particularly important to establish whether the benefits seen in this and in other studies^11^ can be replicated in wider clinical practice.

Finally, in this home-based prehabilitation program, measurements of physical activity and adherence were self-reported, and their accuracy cannot be verified. The use of eHealth technology and activity trackers may provide a more accurate assessment of exercise volume, and this is an area of ongoing research.

In view of our findings in this exploratory study, further research is needed to delineate the relationship between changes in body composition during prehabilitation and clinical outcomes. By limiting the loss of skeletal mass and promoting the loss of VAT before surgery, prehabilitation may have multiple beneficial effects on body composition in esophageal cancer patients.

## Author contributions

LJH, VWB and KM conceived and designed the study; LJH, PRB and ED collected data; LJH, ED and JPB analyzed and interpreted data; LJH and PB drafted the paper; all authors commented upon and revised the paper and all have approved the final draft of the manuscript.

## Supplementary Material

Supplementary_file_1_doac046

Supplementary_file_2_doac046

Supplementary_file_3_doac046

## References

[1] Boshier P R , Heneghan R, Markar S R, Baracos V E, Low D E. Assessment of body composition and sarcopenia in patients with esophageal cancer: a systematic review and meta-analysis. Dis Esophagus 2018; 31(8):1–11. 10.1093/dote/doy047. 29846548

[2] Elliott J A , Doyle S L, Murphy C F et al. Sarcopenia: prevalence, and impact on operative and oncologic outcomes in the multimodal management of locally advanced esophageal cancer. Ann Surg 2017; 266(5): 822–30. 28796017 10.1097/SLA.0000000000002398

[3] Yip C , Goh V, Davies A et al. Assessment of sarcopenia and changes in body composition after neoadjuvant chemotherapy and associations with clinical outcomes in oesophageal cancer. Eur Radiol 2014; 24(5): 998–1005. 24535076 10.1007/s00330-014-3110-4

[4] Anandavadivelan P , Lagergren P. Cachexia in patients with oesophageal cancer. Nat Rev Clin Oncol 2016; 13: 185–96. 26573424 10.1038/nrclinonc.2015.200

[5] Tan B H L , Brammer K, Randhawa N et al. Sarcopenia is associated with toxicity in patients undergoing neo-adjuvant chemotherapy for oesophago-gastric cancer. Eur J Surg Oncol 2015; 41(3): 333–8. 25498359 10.1016/j.ejso.2014.11.040

[6] Makiura D , Ono R, Inoue J et al. Preoperative sarcopenia is a predictor of postoperative pulmonary complications in esophageal cancer following esophagectomy: a retrospective cohort study. J Geriatr Oncol 2016; 7(6): 430–6. 27452909 10.1016/j.jgo.2016.07.003

[7] Carli F , Bessissow A, Awasthi R et al. Prehabilitation: finally utilizing frailty screening data. Eur J Surg Oncol 2020; 46(3): 321–5. 31954550 10.1016/j.ejso.2020.01.001

[8] Moran J , Guinan E, Mccormick P et al. The ability of prehabilitation to influence postoperative outcome after intra- abdominal operation: a systematic review and meta-analysis. Surgery 2016; 160(5): 1189–201. 27397681 10.1016/j.surg.2016.05.014

[9] Hughes M , Hackney R, Lamb P et al. Prehabilitation before major abdominal surgery: a systematic review and meta-analysis. World J Surg 2019; 43(7): 1661–8. 30788536 10.1007/s00268-019-04950-y

[10] Mayanagi S , Tsubosa Y, Omae K et al. Negative impact of skeletal muscle wasting after neoadjuvant chemotherapy followed by surgery on survival for patients with thoracic esophageal cancer. Ann Surg Oncol 2017; 24(12): 3741–7. 28861809 10.1245/s10434-017-6020-2 PMC5658455

[11] Allen S K , Brown V, White D et al. Multimodal prehabilitation during neoadjuvant therapy prior to esophagogastric cancer resection: effect on cardiopulmonary exercise test performance, muscle mass and quality of life: a pilot randomized clinical trial. Ann Surg Oncol 2021; 29: 1839–50. 34725764 10.1245/s10434-021-11002-0

[12] Moug S J , Barry S J E, Maguire S et al. Does prehabilitation modify muscle mass in patients with rectal cancer undergoing neoadjuvant therapy? A subanalysis from the REx randomised controlled trial. Tech Coloproctol 2020; 24(9): 959–64. 32564236 10.1007/s10151-020-02262-1 PMC7429543

[13] Halliday L J , Doganay E, Wynter-Blyth V, Osborn H, Buckley J, Moorthy K. Adherence to pre-operative exercise and the response to prehabilitation in oesophageal cancer patients. J Gastrointest Surg 2020; 25: 890–9. 32314231 10.1007/s11605-020-04561-2 PMC8007503

[14] American College of Sports Medicine . ACSMs Guidelines for Exercise Testing and Prescription. Philadelphia: Lippincott, Williams & Wilkins, 2010.

[15] World Health Organisation . Global Recommendations on Physical Activity for Health. Geneva: World Health Organisation, Geneva, 2010.

[16] Colberg S R , Swain D P, Vinik A I. Use of heart rate reserve and rating of perceived exertion to prescribe exercise intensity in diabetic autonomic neuropathy. Diabetes Care 2003; 26(4): 986–90. 12663561 10.2337/diacare.26.4.986

[17] Borg G . Borg's Perceived Exertion and Pain Scales. Illinois: Human Kinetics, 1998.

[18] Buckley J , Jones J. Tables for Assessing, Monitoring and Guiding Physical Activity/Exercise Intensity in Programmes for Cardiovascular Disease Prevention and Rehabilitation. London: British Association for Cardiovascular Prevention and Rehabilitation, 2012.

[19] Buckley J , Holmes J, Mapp G. Exercise on Prescription: Activity for Cardiovascular Health. Oxford: Butterworth-Heinemann, 1998.

[20] Sykes K , Roberts A. The Chester step test—a simple yet effective tool for the prediction of aerobic capacity. Physiotherapy 2004; 90(4): 183–8.

[21] Hawley-Hague H , Horne M, Skelton D A, Todd C. Review of how we should define (and measure) adherence in studies examining older adults' participation in exercise classes. BMJ Open 2016; 6: e011560. 10.1136/bmjopen-2016-011560 PMC4932302 27338884

[22] Muscaritoli M , Arends J, Bachmann P et al. ESPEN practical guideline: clinical nutrition in cancer. Clin Nutr 2021; 40(5): 2898–913. 33946039 10.1016/j.clnu.2021.02.005

[23] Prado C M M , Lieffers J R, Mccargar L J et al. Prevalence and clinical implications of sarcopenic obesity in patients with solid tumours of the respiratory and gastrointestinal tracts: a population-based study. Lancet Oncol 2008; 9(7): 629–35. 18539529 10.1016/S1470-2045(08)70153-0

[24] Norman K , Stobäus N, Gonzalez M C et al. Hand grip strength: outcome predictor and marker of nutritional status. Clin Nutr 2011; 30(2): 135–42. 21035927 10.1016/j.clnu.2010.09.010

[25] Innes E . Handgrip strength testing: a review of the literature. Aust Occup Ther J 1999; 46(3): 120–40.

[26] Low D E , Alderson D, Cecconello I et al. International consensus on standardization of data collection for complications associated with Esophagectomy: Esophagectomy Complications Consensus Group (ECCG). Ann Surg 2015; 262(2): 286–94. 25607756 10.1097/SLA.0000000000001098

[27] American Thorax Society . Guidelines for the management of adults with hospital-acquired, ventilator-associated, and healthcare-associated pneumonia. Am J Respir Crit Care Med 2005; 171(4): 388–416. 15699079 10.1164/rccm.200405-644ST

[28] Gillis C , Fenton T R, Sajobi T T et al. Trimodal prehabilitation for colorectal surgery attenuates post-surgical losses in lean body mass: a pooled analysis of randomized controlled trials. Clin Nutr 2019; 38(3): 1053–60. 30025745 10.1016/j.clnu.2018.06.982

[29] Yamamoto K , Nagatsuma Y, Fukuda Y et al. Effectiveness of a preoperative exercise and nutritional support program for elderly sarcopenic patients with gastric cancer. Gastric Cancer 2016; 20(5): 913–8. 28032232 10.1007/s10120-016-0683-4

[30] Lagergren J . Influence of obesity on the risk of esophageal disorders. Nat Rev Gastroenterol Hepatol 2011; 8(6): 340–7. 21643038 10.1038/nrgastro.2011.73

[31] Sogabe M , Okahisa T, Kimura T et al. Influence of metabolic syndrome on upper gastrointestinal disease. Clin J Gastroenterol 2016; 9(4): 191–202. 27372302 10.1007/s12328-016-0668-1

[32] Di Caro S , Cheung W H, Fini L et al. Role of body composition and metabolic profile in Barrett’s oesophagus and progression to cancer. Eur J Gastroenterol Hepatol 2016; 28(3): 251–60. 26671515 10.1097/MEG.0000000000000536 PMC4739314

[33] Okumura S , Kaido T, Hamaguchi Y et al. Visceral adiposity and sarcopenic visceral obesity are associated with poor prognosis after resection of pancreatic cancer. Ann Surg Oncol 2017; 24(12): 3732–40. 28871520 10.1245/s10434-017-6077-y

[34] Huang D , Zhou C, Wang S et al. Impact of different sarcopenia stages on the postoperative outcomes after radical gastrectomy for gastric cancer. Surgery 2017; 161(3): 680–93. 27712873 10.1016/j.surg.2016.08.030

[35] Hagens E R C , Feenstra M L, van Egmond M A et al. Influence of body composition and muscle strength on outcomes after multimodal esophageal cancer treatment. J Cachexia Sarcopenia Muscle 2020; 11: 756–67. 32096923 10.1002/jcsm.12540 PMC7296271

[36] Himbert C , Delphan M, Scherer D et al. Signals from the adipose microenvironment and the obesity-cancer link-a systematic review. Cancer Prev Res 2017; 10(9): 494–506. 10.1158/1940-6207.CAPR-16-0322 PMC5898450 28864539

[37] Carli F . Physiologic considerations of enhanced recovery after surgery programs: implications of the stress response. Can J Anesth 2015; 62(2): 110–9. 25501695 10.1007/s12630-014-0264-0

[38] Hopkins B D , Goncalves M D, Cantley L C. Obesity and cancer mechanisms: cancer metabolism. J Clin Oncol 2016; 34(35): 4277–83. 27903152 10.1200/JCO.2016.67.9712 PMC5562429

[39] McTiernan A . Mechanisms linking physical activity with cancer. Nat Rev Cancer 2008; 8(3): 205–11. 18235448 10.1038/nrc2325

[40] Vermillion S A , James A, Dorrell R D et al. Preoperative exercise therapy for gastrointestinal cancer patients: a systematic review. Syst Rev 2018; 7(1):103. 10.1186/s13643-018-0771-0. PMC6058356 30041694

[41] Vissers D , Hens W, Taeymans J et al. The effect of exercise on visceral adipose tissue in overweight adults: a systematic review and meta-analysis. PLoS One 2013; 8(2): e56415. 10.1371/journal.pone.0056415. PMC3568069 23409182

[42] Maillard F , Pereira B, Boisseau N. Effect of high-intensity interval training on total, abdominal and visceral fat mass: a meta-analysis. Sports Med 2017; 48(2): 269–88. 10.1007/s40279-017-0807-y 29127602

[43] Devin J L , Jenkins D G, Sax A T et al. Cardiorespiratory fitness and body composition responses to different intensities and frequencies of exercise training in colorectal cancer survivors. Clin Colorectal Cancer 2018; 17(2): e269–79. 29397328 10.1016/j.clcc.2018.01.004

[44] Toohey K , Pumpa K L, Arnolda L, Cooke J, Yip D, Craft P S, Semple S. A pilot study examining the effects of low-volume high-intensity interval training and continuous low to moderate intensity training on quality of life, functional capacity and cardiovascular risk factors in cancer survivors. PeerJ 2016; 4: e2613. 27781180 10.7717/peerj.2613 PMC5075690

[45] Palma S , Hasenoehrl T, Jordakieva G et al. High-intensity interval training in the prehabilitation of cancer patients: a systematic review and meta-analysis. Support Care Cancer 2020; 29(4): 1781–94. 33106975 10.1007/s00520-020-05834-x PMC7892520

[46] Thompson D , Karpe F, Lafontan M et al. Physical activity and exercise in the regulation of human adipose tissue physiology. Physiol Rev 2012; 92(1): 157–91. 22298655 10.1152/physrev.00012.2011

[47] Verheggen R J H M , Maessen M F H, Green D J et al. A systematic review and meta-analysis on the effects of exercise training versus hypocaloric diet: distinct effects on body weight and visceral adipose tissue: effects of exercise versus diet on visceral fat. Obes Rev 2016; 17(8): 664–90. 27213481 10.1111/obr.12406

[48] Macmillan Cancer Support . Prehabilitation for people with cancer: principles and guidance for prehabilitation within the management and support of people with cancer*.* Available from: https://www.macmillan.org.uk/healthcare-professionals/news-and-resources/guides/principles-and-guidance-for-prehabilitation [Accessed 10 December 2020].

