# Supplementary material for: The effects of prehabilitation on body composition in patients undergoing multimodal therapy for esophageal cancer
Source: Dis Esophagus. 2022 Jul 7;36(2):doac046. doi: 10.1093/dote/doac046 (PMC9885737; doi:10.1093/dote/doac046)
Supplement: Supplementary_file_1_doac046 [file supplementary_file_1_doac046.docx]

**Supplementary file 1** – example of a personalised exercise prescription

**exercise prescription card**

**Name: Week Number: Date at start of the week:**

| **Activity** |  | **Monday** | **Tuesday** | **Wednesday** | **Thursday** | **Friday** | **Saturday** | **Sunday** |
| --- | --- | --- | --- | --- | --- | --- | --- | --- |
| **Bike**  15mins, 5x a week  RPE 14 | Duration |  |  |  |  |  |  |  |
|  | Intensity RPE |  |  |  |  |  |  |  |
| **Walking**  30mins, 5x a week  RPE 14 | Duration |  |  |  |  |  |  |  |
|  | Intensity RPE |  |  |  |  |  |  |  |
| **Seat to stand**  2x 15 reps, 5x a week | Duration |  |  |  |  |  |  |  |
|  | Intensity RPE |  |  |  |  |  |  |  |
| **Squat**  3x 15 reps, 5x a week | Duration |  |  |  |  |  |  |  |
|  | Intensity RPE |  |  |  |  |  |  |  |
| **Stair climbing**  6 mins, 5x a week | Duration |  |  |  |  |  |  |  |
|  | Intensity RPE |  |  |  |  |  |  |  |
| **Press ups**  3 x 15 reps, 5x a week | Duration |  |  |  |  |  |  |  |
|  | Intensity RPE |  |  |  |  |  |  |  |
