# Supplementary material for: The effects of prehabilitation on body composition in patients undergoing multimodal therapy for esophageal cancer
Source: Dis Esophagus. 2022 Jul 7;36(2):doac046. doi: 10.1093/dote/doac046 (PMC9885737; doi:10.1093/dote/doac046)
Supplement: Supplementary_file_2_doac046 [file supplementary_file_2_doac046.docx]

**Supplementary file 2 – propensity score matched comparison**

Table A. Baseline comparison of propensity score matched groups

|  | Prehabilitation patients  (n=28) | Controls  (n=28) | p value |
| --- | --- | --- | --- |
| Age (years) | 65.3 ± 9.6 | 63.5 ± 9.6 | 0.496 |
| Gender, male n (%) | 21 (75%) | 22 (76%) | 0.752 |
| Tumour location n (%) |  |  |  |
| Oesophagus | 17 (61%) | 17 (61%) | 0.999 |
| GOJ | 11 (39%) | 11 (39%) |  |
| Histological subtype n (%) |  |  |  |
| Adenocarcinoma | 25 (89%) | 21 (75%) | 0.163 |
| Squamous cell carcinoma | 3 (11%) | 7 (25%) |  |
| Neoadjuvant therapy n (%) |  |  |  |
| Chemotherapy | 22 (79%) | 21 (75%) | 0.752 |
| Chemoradiotherapy | 6 (21%) | 7 (25%) |  |
| Clinical stage n (%) |  |  |  |
| I | 1 (3%) | 1 (3%) | 0.852 |
| II | 3 (11%) | 3 (11%) |  |
| II | 18 (64%) | 15 (54%) |  |
| IV | 6 (22%) | 9 (32%) |  |
| ASA grade n (%) |  |  |  |
| II | 25 (89%) | 21 (75%) | 0.163 |
| III | 3 (11%) | 7 (25%) |  |
| Charlson Comorbidity Index | 4.8 ± 1.5 | 4.4 ± 1.2 | 0.371 |
| Time interval between scans days | 98.7 ± 22.9 | 119.0 ± 39.5 | 0.023 |
| Oesophagectomy |  |  |  |
| 2-stage | 35 (68%) | 15 (54%) | 0.314 |
| 3-stage | 9 (18%) | 9 (32%) |  |
| Thoracoabdominal | 7 (14%) | 4 (14%) |  |
| Surgical approach |  |  |  |
| Open | 27 (96%) | 25 (89%) | 0.299 |
| *Hybrid minimally invasive* † | 1 (4%) | 3 (11%) |  |
| Baseline body composition |  |  |  |
| Weight (kg) | 84.9 ± 18.2 | 79.5 ± 13.3 | 0.206 |
| BMI (kg/m^2^) | 28.6 ± 5.2 | 26.6 ± 3.5 | 0.105 |
| SM area (cm^2^) | 143.6 ± 31.8 | 149.3 ± 26.7 | 0.467 |
| SMI (cm^2^/m^2^) | 48.0 ± 7.3 | 49.9 ± 6.8 | 0.314 |
| TAT area (cm^2^) | 421.8 ± 168.3 | 403.6 ± 154.4 | 0.675 |
| VAT area (cm^2^) | 181.6 ± 87.3 | 176.0 ± 102.7 | 0.829 |
| SAT area (cm^2^) ‡ | 224.2 (173.3, 281.8) | 211.1 (165.1, 296.4) | 0.831 |
| Sarcopenia, n (%) | 17 (61%) | 14 (50%) | 0.420 |
| Visceral obesity, n (%) | 19 (68%) | 15 (54%) | 0.274 |
| *Continuous data presented as mean ± SD unless otherwise stated. GOJ = gastro oesophageal junction; ASA = American Society of Anesthesiologist physical status classification; SM = skeletal muscle; SMI = skeletal muscle index; TAT = total adipose tissue; VAT = visceral adipose tissue; SAT = subcutaneous adipose tissue; n/a = not applicable.* †*Laparoscopic abdominal stage, open thoracic stage.* ‡*Non-parametric data, displayed as median (interquartile range)* | | | |

Table B. Propensity score matched comparison of change in body composition parameters during neoadjuvant therapy

|  | **Prehabilitation patients**  **(n=28)** | **Controls**  **(n=28)** | **Mean difference**  **(95% CI)** | **p value** |
| --- | --- | --- | --- | --- |
| **Δ Weight (kg)** | -1.5 ± 5.3 | -3.0 ± 4.5 | -1.5 (-4.2 to 1.1) | 0.248 |
| **Δ BMI (kg/m^2^)** | -0.5 ± 1.7 | -1.1 ± 1.5 | -0.5 (-1.4 to 0.3) | 0.221 |
| **Δ SM area (cm^2^)** | -9.1 ± 10.9 | -15.5 ± 11.3 | -6.4 (-12.4 to -0.5) | 0.035 |
| **Δ SMI (cm^2^/m^2^)** | -2.9 ± 3.5 | -5.2 ± 3.7 | -2.3 (-4.3 to -0.3) | 0.023 |
| **Δ TAT area (cm^2^)** | -30.3 ± 72.7 | -33.3 ± 63.2 | -3.0 (-39.5 to 33.5) | 0.868 |
| **Δ VAT area (cm^2^)** | -14.5 ± 41.7 | -19.4 ± 42.4 | -4.9 (-27.5 to 17.6) | 0.664 |
| **Δ SAT area (cm^2^)** † | -12.2 (-29.7, 9.1) | -11.0 (-35.7, 4.8) | n/a | 0.987 |
| **Relative Δ Weight (%)** | -1.4 ± 5.7 | -3.9 ± 5.9 | -2.5 (-5.6 to 0.6) | 0.110 |
| **Relative Δ BMI (%)** | -1.4 ± 5.7 | -3.9 ± 5.9 | -2.5 (-5.6 to 0.6) | 0.110 |
| **Relative Δ SM area (%)** | -5.9 ± 7.3 | -10.6 ± 7.5 | -4.7 (-8.6 to -0.7) | 0.021 |
| **Relative Δ SMI (%)** | -5.9 ± 7.3 | -10.6 ± 7.5 | -4.7 (-8.6 to -0.7) | 0.021 |
| **Relative Δ TAT area (%)** † | -7.1 (-15.7, 3.9) | -9.4 (-19.8, -0.1) | n/a | 0.670 |
| **Relative Δ VAT area (%)** † | -11.3 (-23.8, 2.7) | -12.4 (-30.5, 0.9) | n/a | 0.629 |
| **Relative Δ SAT area (%)** † | -5.2 (-15.5, 5.5) | -5.8 (-14.9, 1.6) | n/a | 0.806 |
| *Continuous data presented as mean ± SD unless otherwise stated. SM = skeletal muscle; SMI = skeletal muscle index; TAT = total adipose tissue; VAT = visceral adipose tissue; SAT = subcutaneous adipose tissue; n/a = not applicable.* †*Non-parametric data, displayed as median (interquartile range)* | | | | |

Table C. Propensity score matched comparison of post-operative complications

|  | Prehabilitation patients  (n=28) | Controls  (n=28) | p value |
| --- | --- | --- | --- |
| Any complication n (%) | 17 (61%) | 20 (71%) | 0.397 |
| Severe complications n (%) † | 8 (29%) | 14 (50%) | 0.101 |
| Lower respiratory tract infection  n (%) | 10 (36%) | 15 (54%) | 0.179 |
| † *Clavien Dindo Grade 3 or higher* | | | |
