# Supplementary material for: The effects of prehabilitation on body composition in patients undergoing multimodal therapy for esophageal cancer
Source: Dis Esophagus. 2022 Jul 7;36(2):doac046. doi: 10.1093/dote/doac046 (PMC9885737; doi:10.1093/dote/doac046)
Supplement: Supplementary_file_3_doac046 [file supplementary_file_3_doac046.docx]

**Supplementary file 3 – multivariate analyses**

Table A. Factors associated with loss of skeletal mass and loss of visceral adipose tissue during prehabilitation

|  | **B coefficient (95% CI)** | **SE** | **p value** |
| --- | --- | --- | --- |
| **Loss of skeletal mass** |  |  |  |
| Age | 0.52 (-0.13 to 1.17) | 0.32 | 0.110 |
| ASA | 7.62 (-5.39 to 20.62) | 6.37 | 0.241 |
| CCI | -1.06 (-6.51 to 4.39) | 2.67 | 0.693 |
| Clinical stage | 5.44 (-3.61 to 14.49) | 4.43 | 0.229 |
| Adherence (%) | -13.54 (-39.21 to 12.14) | 12.57 | 0.290 |
| Average PA (MET min wk^-1^) | 0.01 (-0.01 to 0.02) | 0.01 | 0.463 |
| **Loss of visceral adipose tissue** | | | |
| Age | 0.44 (-1.94 to 2.81) | 1.16 | 0.709 |
| ASA | 36.78 (-10.96 to 84.52) | 23.37 | 0.126 |
| CCI | -8.98 (-28.98 to 11.02) | 9.79 | 0.367 |
| Clinical stage | -9.02 (-42.24 to 24.20) | 16.27 | 0.583 |
| Adherence (%) | -81.46 (-175.70 to 12.79) | 46.15 | 0.088 |
| Average PA (MET min wk^-1^) | 0.04 (0.01 to 0.07) | 0.02 | 0.046 |
| *CCI = Charlson Comorbidity Index; PA = physical activity* | | | |

Table B. Factors associated with post-operative complications

| **Variable** | **Odds ratio (95% CI)** | **SE** | **p value** |
| --- | --- | --- | --- |
| Age | 1.00 (0.91 to 1.09) | 0.01 | 0.948 |
| ASA | 6.58 (0.98 to 44.04) | 0.97 | 0.052 |
| CCI | 0.93 (0.44 to 1.96) | 0.38 | 0.854 |
| Clinical stage III † | 1.07 (0.17 to 6.68) | 0.93 | 0.939 |
| Clinical stage IV † | 1.31 (0.35 to 4.85) | 0.67 | 0.685 |
| SM loss (cm^2^/m^2^) | 1.00 (0.97 to 1.05) | 0.02 | 0.761 |
| VAT loss (cm^2^) | 0.98 (0.96 to 0.99) | 0.01 | 0.017 |
| *CCI = Charlson Comorbidity Index; SM= skeletal muscle; VAT = visceral adipose tissue.*  † *Clinical stage compared with stage II* | | | |
